# Modes of Action of a Bi-domain Plant Defensin MtDef5 Against a Bacterial Pathogen *Xanthomonas campestris*

**DOI:** 10.3389/fmicb.2018.00934

**Published:** 2018-05-16

**Authors:** Siva L. S. Velivelli, Kazi T. Islam, Eric Hobson, Dilip M. Shah

**Affiliations:** ^1^Donald Danforth Plant Science Center, St. Louis, MO, United States; ^2^Department of Biology, Jackson State University, Jackson, MS, United States

**Keywords:** antimicrobial peptides, antibacterial defensin, membrane permeabilization, *Xanthomonas campestris*, *Clavibacter michiganensis*, *Medicago truncatula*

## Abstract

Defensins are small cysteine-rich endogenous host defense peptides expressed in all higher plants. They are thought to be important players in the defense arsenal of plants against fungal and oomycete pathogens. However, little is known regarding the antibacterial activity of these peptides. The genome of the model legume *Medicago truncatula* contains 63 genes each encoding a defensin with a tetradisulfide array. A unique bi-domain defensin, designated MtDef5, was recently characterized for its potent broad-spectrum antifungal activity. This 107-amino acid defensin contains two domains, 50 amino acids each, linked by a short peptide APKKVEP. Here, we characterize antibacterial activity of this defensin and its two domains, MtDef5A and MtDef5B, against two economically important plant bacterial pathogens, Gram-negative *Xanthomonas campestris* and Gram-positive *Clavibacter michiganensis*. MtDef5 inhibits the growth of *X. campestris*, but not *C. michiganensis*, at micromolar concentrations. MtDef5B, but not MtDef5A, exhibits more potent antibacterial activity than its parent MtDef5. MtDef5 and each of its two domains induce distinct morphological changes and cell death in *X. campestris*. They permeabilize the bacterial plasma membrane and translocate across membranes to the cytoplasm. They bind to negatively charged DNA indicating these peptides may kill bacterial cells by inhibiting DNA synthesis and/or transcription. The cationic amino acids present in the two γ-core motifs of MtDef5 that were previously shown to be important for its antifungal activity are also important for its antibacterial activity. MtDef5 and its more potent single domain MtDef5B have the potential to be deployed as antibacterial agents for control of a Xanthomonas wilt disease in transgenic crops.

## Introduction

Plants possess a sophisticated innate immune system to counter pathogenic attack. They produce several antimicrobial peptide (AMP) families via their canonical ribosomal gene expression machinery (Goyal and Mattoo, 2014; Breen et al., 2015; Tavormina et al., 2015). Defensins are small cationic cysteine-rich AMPs that are ubiquitous in higher plants and contribute to their innate immunity. Genes encoding antifungal defensins have been widely used for engineering broad-spectrum resistance to fungal pathogens in transgenic crops (Kaur et al., 2011; De Coninck et al., 2013). Based on their predicted subcellular localization, plant defensins are designated as class I or class II. Class I defensins are synthesized as precursor proteins comprising the secretory signal peptide and the mature domain. The precursor defensins enter the secretory pathway where they are proteolytically processed into active mature peptides and released into apoplast. Class II defensins are synthesized as larger precursors containing an additional carboxy-terminal propeptide sequence of 27–33 amino acids and are targeted to the vacuole (Lay et al., 2014). The three-dimensional protein structure of plant defensins is highly conserved and comprises one α-helix and three antiparallel β-strands that are internally stabilized by four disulfide cross-links. Despite their structural conservation, plant defensins are diverse in their amino acid sequences. The sequence diversity contributes to a variety of biological functions attributed to these peptides (Carvalho Ade and Gomes, 2009; Sagaram et al., 2013; Vriens et al., 2014).

Past studies on plant defensins have primarily focused on their antifungal activity, with relatively few studies addressing their antibacterial activity. These peptides have been extensively characterized for their ability to inhibit the growth of fungal and oomycete pathogens *in vitro* and in plants (Kaur et al., 2011; De Coninck et al., 2013; Cools et al., 2017; Parisi et al., 2018). However, the modes of action (MOA) of only a few antifungal defensins have been studied in detail (Cools et al., 2017; Parisi et al., 2018). Very few defensins with antibacterial activity have been reported (van der Weerden and Anderson, 2013). For example, Cp-thionin from cowpea (Franco et al., 2006; Kraszewska et al., 2016), DmAMP1 from *Dahlia merckii*, CtAMP1 from *Clitoria ternatea*, AhAMP1 from *Aesculus hippocastanum* (Osborn et al., 1995), *Zm*ESR-6 from maize (Balandin et al., 2005), fabatin-2 from broad bean (Zhang and Lewis, 1997; Kraszewska et al., 2016), and SOD1-7 from spinach (Segura et al., 1998) have been reported to exhibit antibacterial activity against a range of Gram-positive and Gram-negative bacterial pathogens. Among antibacterial defensins, only SOD2 from spinach has been demonstrated to confer resistance to Asiatic citrus canker (ACC) and Huanglongbing (HLB) caused by *Xanthomonas citri* ssp. *citri* and *Candidatus Liberibacter* sp., respectively, in transgenic citrus (Stover et al., 2013). The MOA of antibacterial plant defensins have yet to be deciphered in detail.

*Medicago truncatula* genome contains 63 genes each encoding a defensin with four disulfide bonds (Maróti et al., 2015). One of these genes encodes a bi-domain defensin MtDef5 containing two defensin domains, 50 amino acids each, connected by a 7-amino acid linker sequence APKKVEP. This defensin is predicted to be targeted to the apoplast. Recently, we have reported the potent antifungal activity and MOA of MtDef5 (Islam et al., 2017). Here, we report the antibacterial activity and MOA of this defensin and its two domains, MtDef5A and MtDef5B. We show that MtDef5 inhibits the growth of the Gram-negative bacterial pathogen *X. campestris* pv. *campestris* 8004, but not the Gram-positive bacterial pathogen *Clavibacte*r *michiganensis* subsp. *Nebraskensis* strain CIC 395. The single domain MtDef5B, but not MtDef5A, exhibits more potent antibacterial activity against *X. campestris* pv. *campestris* 8004 than MtDef5. The MOA studies show that MtDef5 and MtDef5B are both translocated into the cells of *X. campestris*, but induce different morphological changes and bind to negatively charged DNA *in vitro*. Using site-directed mutagenesis, we demonstrate that cationic amino acid residues present in each of the two γ-core motifs of MtDef5 are critical for its antibacterial activity.

## Materials and Methods

### Bacterial Strains and Growth Conditions

The Gram-positive pathogenic bacterial strain, *Clavibacte*r *michiganensis* subsp. *Nebraskensis* strain CIC 395 (hereafter referred to as *C. michiganensis*) and the model Gram-negative pathogenic bacterial strain, *Xanthomonas campestris* pv. *campestris* 8004 (hereafter referred to as *X. campestris*), were kindly provided by Dr. Dean Malvick of the University of Minnesota, MN and Dr. Rebecca Bart of the Donald Danforth Plant Science Center, St. Louis, MO, United States, respectively. These two bacterial pathogens are economically important pathogens of plants (Mansfield et al., 2012; Saleem et al., 2017). *C. michiganensis* and *X. campestris* were routinely grown at 28 ± 2°C for 2–3 days on nutrient broth-yeast extract agar (NBY, 8 g/l nutrient broth, 2 g/l yeast extract, 2.5 g/l glucose and 2 g/l K_2_HPO_4_, 0.5 g/l KH_2_PO_4_, 1 mM MgSO_4_, 15 g/l agar) containing cycloheximide (75 μg ml^-1^) and nutrient-yeast extract glycerol agar (NYGA, 5 g/l bacto peptone, 3 g/l yeast extract, 20 ml glycerol, 10 g/l agar) containing rifampicin (100 μg ml^-1^), respectively.

### Expression and Purification of Defensins

The chemically synthesized MtDef5A with four disulfide bonds was obtained from JPT Peptide Technologies (Berlin, Germany). MtDef5, MtDef5B, and MtDef5_V1 (MtDef5 H36A, R37A/H93A, R94A) were generated by recombinant expression in *Pichia pastoris* and purified using the CM-Sephadex C-25 cation-exchange chromatography as described previously (Islam et al., 2017).

### Antibacterial Assay

The antibacterial activity of MtDef5, MtDef5_V1, MtDef5A, and MtDef5B against *X. campestris* and *C. michiganensis* was determined as described previously with minor modifications (Balandin et al., 2005; Farkas et al., 2017). A single colony of each bacterial strain was inoculated into NBY and NYG broth, respectively, and grown overnight at 28 ± 2°C on a rotary shaker at 225 rpm. Bacterial cells were diluted to 2 × 10^4^ cfu/ml in 2X NBY and NYG broth, respectively. The antibacterial assay was performed in a 0.5 ml eppendorf tube, in which 50 μl of 2 × 10^4^ cfu/ml bacterial inoculum corresponding to a final test concentration of 1 × 10^4^ cfu/ml was incubated with 50 μl of the various concentrations (0.75–12 μM) of each defensin at 28 ± 2°C on a rotary shaker at 125 rpm for 48 h. After incubation, 10 μl of 0.1% resazurin solution (Sigma-Aldrich, United States) was added to each tube and re-incubated overnight. A change from blue to pink color indicates reduction of resazurin and the lowest concentration of peptide at which no color change is observed was determined as minimal inhibitory concentration (MIC). For determination of minimum bactericidal concentration (MBC), 100 μl aliquot from tubes showing no visible bacterial growth was plated and incubated at 28 ± 2°C for 24–48 h. The lowest concentration of peptide at which a 99.9% reduction in the initial microbial inoculum occurred was defined as MBC.

### Membrane Permeability Assay

The effect of defensins on the membrane integrity of *X. campestris* was determined by using the propidium iodide (PI) uptake assay, as described previously, with minor modifications (Stiefel et al., 2015). The assay was performed in a 0.5 ml eppendorf tube, in which 50 μl of 2 × 10^7^ cfu/ml bacterial inoculum corresponding to a final test concentration of 1 × 10^7^ cfu/ml was incubated with 50 μl of each defensin at MIC at 28 ± 2°C on a rotary shaker at 125 rpm for 3 h. After incubation, PI (10 μg/ml final concentration) was added to each tube. After 20 min of incubation in dark, the cells were placed in 10 mm microwell of 35 mm glass bottom dishes (MatTek Corporation, Ashland, MA, United States) and imaged using a Leica SP8-X confocal microscope (63× magnification) with an excitation/emission wavelength of 543/555 nm, respectively.

### Internalization and Subcellular Localization of MtDef5 and MtDef5B

MtDef5 and MtDef5B were each labeled with DyLight550 amine-reactive dye according to the manufacturer’s instructions (Thermo Scientific, United States). The assay was performed in 10 mm microwell of 35 mm glass bottom microwell dish (MatTek Corporation, Ashland, MA, United States). The *X. campestris* bacterial cells (final test concentration of 1 × 10^7^ cfu/ml) were either treated with DyLight550-MtDef5 or DyLight550-MtDef5B (1 × MIC) and co-stained with nucleic acid selective dye SYTOX Green (SG) (1 μM) and incubated in dark at 28 ± 2°C on a rotary shaker at 125 rpm for different time points (5–60 min). Internalization and subcellular localization of each DyLight550-labeled defensin were visualized using the Leica SP8-X confocal microscope (100× magnification). The DyLight550-labeled defensin was excited at 550 nm and fluorescence was detected at 560–600 nm, whereas SG was excited at 488 nm and fluorescence was detected at 510–530 nm.

### DNA–Defensin Interaction Assay

Gel retardation assay was performed as described previously with minor modifications (Park et al., 1998; Li et al., 2016; Shi et al., 2016). Genomic DNA was purified from *X. campestris* using a E.Z.N.A. Bacterial DNA Kit *(*Omega Biotek, United States*)*. In addition, pUC57 plasmid DNA containing a naive 132 bp insert was also used. The gel shift experiments were performed by mixing 200 ng of the genomic DNA or plasmid DNA with different concentrations of MtDef5, MtDef5A, MtDef5B, and MtDef5_V1 (0, 3, 6, and 12 μM) in 20 μl of DNA binding buffer (5% glycerol, 10 mM Tris-HCl, pH 8.0, 1 mM EDTA, 1 mM DTT, 20 mM KCl, and 50 μg/ml of BSA). The reaction mixtures were incubated for 1 h at room temperature and then mixed with 2 μl of 6X gel loading dye (B7024S, New England Biolabs, MA, United States). The protein–DNA interaction was visualized using a Bio-Rad ChemiDoc XRS+ system following electrophoresis in 1% agarose gel containing ethidium bromide with 1× TAE buffer at 120V for 45 min.

## Results

### MtDef5 and Its Two Domains Exhibit Potent Antibacterial Activity Against *X. campestris*, but Not Against *C. michiganensis*

**FIGURE 1:**
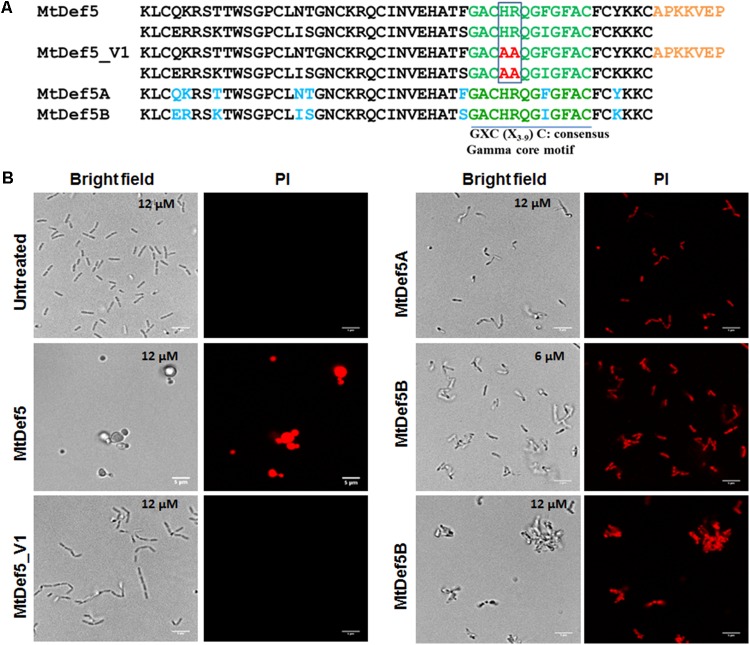
**(A)** The deduced amino acid sequences of MtDef5, MtDef5_V1, MtDef5A, and MtDef5B. The bi-domain MtDef5 protein containing its two defensin domains, MtDef5A and MtDef5B, each 50 amino acids in length, are connected by a 7-amino acid linker shown in orange. Amino acid differences between the two domains are shown in blue. The γ-core motif is shown in green. The cationic amino acid residues histidine and arginine of the two γ-core motifs (highlighted in red) were simultaneously replaced with alanine. **(B)** Disruption of the cell membranes of *X. campestris* cells by MtDef5, MtDef5_V1, MtDef5A, and MtDef5B. Confocal microscopy images of bacterial cells treated with MtDef5, MtDef5A, MtDef5B, or MtDef5_V1 in presence of the membrane impermeant dye PI. Internalization of the red fluorescent PI by the bacterial cells indicates membrane disruption. The bar = 5 μm.

We have previously reported the amino acid sequence and broad spectrum antifungal activity of the bi-domain defensin MtDef5. The amino acid sequence of this defensin is shown in **Figure 1A**. It shows the sequences of its two single domains, MtDef5A and MtDef5B, and the linker sequence connecting them. MtDef5 defensin inhibits the growth of the ascomycete fungi, *Neurospora crassa* and *Fusarium graminearum*, at submicromolar concentrations (Islam et al., 2017). Based on its high cationicity and hydrophobicity, we decided to test its *in vitro* antibacterial activity against the Gram-negative bacterial pathogen *X. campestris* and the Gram-positive bacterial pathogen *C. michiganensis*. MtDef5 and its single domains inhibited the growth of *X. campestris* at micromolar concentrations but with varying potency. Surprisingly, MtDef5B was most active with a MIC of 6 μM and a MBC of 12 μM. The MIC value of MtDef5 and MtDef5A against *X. campestris* was 12 μM, while the MBC value was greater than 12 μM (**Table 1**). No antibacterial activity for MtDef5, MtDef5A, or MtDef5B was observed against *C. michiganensis* even at concentrations exceeding 12 μM. We have previously tested four different γ-core motif variants of MtDef5 for their antifungal activity against a fungal pathogen *F. graminearum* and found that MtDef5_V1 variant carrying the H36A, R37A/H93A, R94A substitutions had lost its antifungal activity (Islam et al., 2017). We therefore decided to test its antibacterial activity against *X. campestris* and *C. michiganensis*. This variant did not inhibit the growth of either bacterial pathogen at concentrations exceeding 12 μM (**Table 1**).

**Table 1:** Minimum inhibitory concentration (MIC) and minimal bactericidal concentration (MBC) of MtDef5, MtDef5A, MtDef5B, and MtDef5_V1 required for antibacterial activity against *Xanthomonas campestris* and *Clavibacter michiganensis.*

MIC and MBC (μM)				
	*X. campestris*		*C. michiganensis*	
	MIC	MBC	MIC	MBC
MtDef5	12	>12	>12	>12
MtDef5 A	12	>12	>12	>12
MtDef5 B	6	12	>12	>12
MtDef5_V1	>12	>12	>12	>12

### MtDef5 and Its Two Domains Disrupt Cell Membrane and Induce Morphological Changes in *X. campestris*

As part of their MOA, cationic antibacterial peptides alter the membrane integrity of their target microbes (Yeaman and Yount, 2003; Bahar and Ren, 2013; Joo et al., 2016). We determined the effect of MtDef5, MtDef5A, MtDef5B, and MtDef5_V1 on membrane permeability using the membrane impermeant fluorescent red dye PI. The PI only penetrates the bacterial cells with damaged membranes and binds to nucleic acids; it is generally excluded from viable cells with intact cell membranes. The uptake of PI was assessed using confocal microscopy in bacterial cells of *X. campestris* treated with MtDef5, MtDef5A, MtDef5B and MtDef5_V1 at their respective MIC values. The uptake of PI was visible in bacterial cells treated with either of these defensins and was indicative of the loss of cell viability. Further, all three defensins induced distinct morphological changes in *X. campestris* cells (**Figure 1B**). The untreated bacteria used as control appear as normal rod-shaped cells and, as expected, did not take up PI. In contrast, in the presence of MtDef5, *X. campestris* cells became spherical or dumbbell-shaped and took up PI indicating cell viability was lost. In presence of MtDef5A, *X. campestris* cells became somewhat condensed and slightly reduced in size and also lost viability as indicated by the uptake of PI. Interestingly, in presence of 6 μM MtDef5B, *X. campestris* cell integrity was severely compromised and cells aggregated into small network-like clusters. At the MBC value of 12 μM, bacterial cells formed even more prominent network-like clusters or aggregations. Interestingly, cells treated with the MtDef5 γ-core motif variant, MtDef5_V1, formed chain-like structures, but remained viable as indicated by the complete absence of PI uptake (**Figure 1B**). The observed changes in the morphology of bacterial cells are apparently specific to each defensin since challenge with 70% isopropanol kills *X. campestris* cells, as shown by the uptake of PI, but does not induce any obvious morphological changes (Supplementary Figure S1). We conclude from these studies that MtDef5 and its two domains induce distinct morphological changes in cells of *X. campestris* and cause cell death. In addition, mutations of the cationic amino acids (H36, R37, H96, and R97) in the two γ-core motifs of MtDef5 lead to a loss of bacterial cell killing indicating importance of these motifs in the antibacterial action of MtDef5.

### MtDef5 and MtDef5B Accumulate in the *X. campestris* Cytoplasm

**FIGURE 2:**
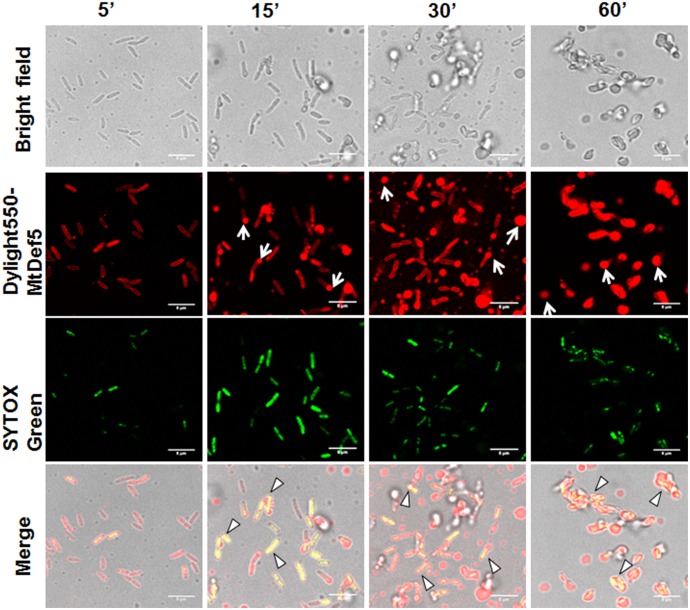
MtDef5 localizes into the cytoplasm of *X. campestris* and induces morphological changes. Confocal microscopy images of bacterial cells treated with DyLight550-labeled MtDef5. MtDef5 first bind to the bacterial surface and subsequently translocates into the cytoplasm. The cells appear as spherical within 60 min (arrows). The co-localization of the DyLight550-labeled MtDef5 and the nucleic acid-complexed SYTOX Green (SG) is clearly visible using confocal microscopy (triangles).

**FIGURE 3:**
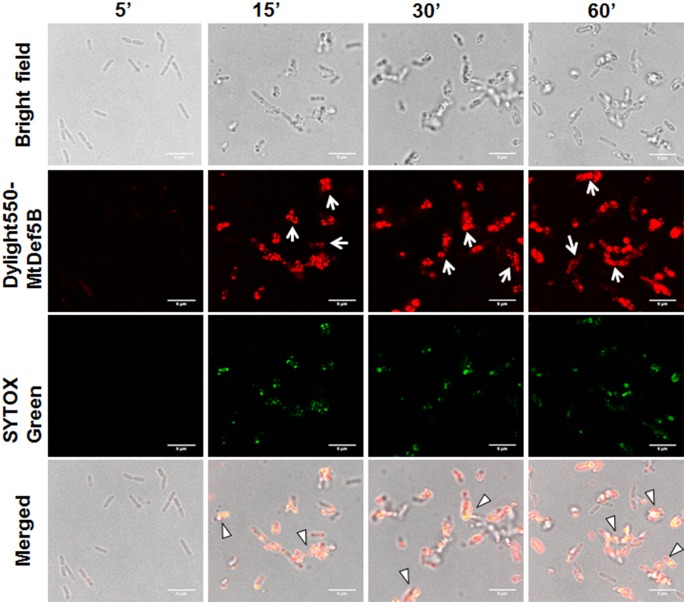
MtDef5B causes significant membrane damage and translocated into the cytoplasm of *X. campestris*. Confocal microscopy images of bacterial cells treated with DyLight550-labeled MtDef5B. MtDef5B binds to the bacterial surface and translocates into the cytoplasm. Significant disruption of the membrane integrity is evident (arrows). The co-localization of the DyLight550-labeled MtDef5 and the nucleic acid-complexed SYTOX Green (SG) is clearly visible using confocal microscopy (triangles).

To gain additional insight into the MOA of these defensins, confocal microscopy was performed on bacterial cells treated with either DyLight550-labeled MtDef5 or MtDef5B. Both MtDef5 and MtDef5B penetrated the cell membrane and accumulated in the cytoplasm of *X. campestris* cells. Within 5 min of treatment, MtDef5 bound to the bacterial surface and subsequently translocated into the cytoplasm of bacterial cells. After 15 min, a small proportion of cells appeared swollen at the poles, and within 30–60 min, the vast majority of cells became spherical or dumbbell-shaped (**Figure 2**). In contrast, MtDef5B caused significant membrane damage to the bacterial cells and accumulated in the cytoplasm of *X. campestris* cells as visualized by confocal microscopy within 15 min of treatment. Furthermore, MtDef5B provoked leakage of cellular content and caused cell aggregation within 30–60 min (**Figure 3**). Intracellular localization of MtDef5 and MtDef5B was further investigated by examining co-localization of DyLight550-labeled MtDef5 or MtDef5B with the membrane permeant dye SG, which binds to nucleic acids and its fluorescence increases >500-fold upon binding. An influx of SG into the cytoplasm of *X. campestris* was observed within 5 min of challenge with MtDef5 and further increased at 15 min of challenge (**Figure 2**). However, cells became less competent to take up SG at later time points. In *X. campestris* cells treated with MtDef5B, no uptake of SG was observed at 5 min and, compared with MtDef5 challenge, much less SG uptake was observed at all time points indicating that membrane permeabilization is perhaps not a major factor contributing to the antibacterial action of MtDef5B (**Figure 3**), albeit with a more pronounced membrane damage.

### MtDef5, MtDef5A, and MtDef5B Bind to DNA

**FIGURE 4:**
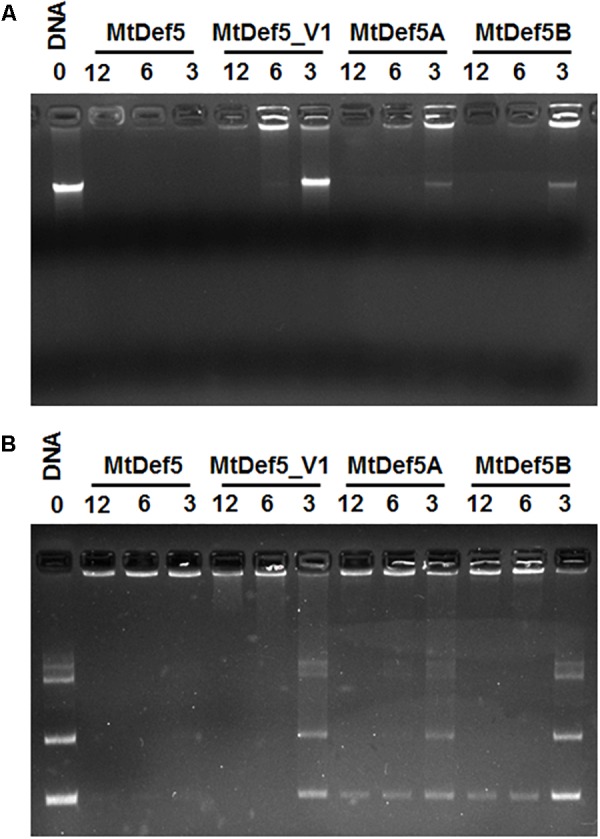
Gel retardation assay showing the binding of MtDef5, MtDef5_V1, MtDef5A, and MtDef5B to *X. campestris* DNA and pUC57 plasmid DNA. **(A)** The binding of various concentrations (0, 3, 6, and 12 μM) of defensins with 200 ng of bacterial DNA. **(B)** The binding of various concentrations of defensins (0, 3, 6, and 12 μM) with 200 ng of pUC57 plasmid DNA carrying a naive 132 bp insert. Note that the wild-type bi-domain MtDef5 binds to DNA at all concentrations tested, whereas MtDef5_V1, MtDef5A, and MtDef5B show weak binding at 3 μM.

Cationic AMPs are known to interact with intracellular targets, such as nucleic acids, as part of their MOA (Park et al., 1998; Shi et al., 2016). To determine if the electrostatic interaction with DNA is one of the factors contributing to the inhibitory activity of MtDef5, MtDef5A, MtDef5B, the *in vitro* DNA binding ability of these peptides was assessed by a gel-retardation assay. As shown in **Figures 4A,B**, MtDef5 bound to *X. campestris* DNA and the plasmid DNA inducing gel retardation at all concentrations tested indicating peptide-induced precipitation of DNA. In contrast, MtDef5_V1 with the H36A, R37A, H93A, and R94A substitutions in its two γ-core motifs bound to DNA at 6 and 12 μM, but at 3 μM its DNA binding ability was markedly reduced. MtDef5A and MtDef5B also bound strongly to *X. campestris* and plasmid DNAs at concentrations of 6 and 12 μM, but at concentration of 3 μM, the DNA-binding of these peptides was significantly reduced. Based on these observations, we propose that the antibacterial MOA of MtDef5, MtDef5A, and MtDef5B likely involves electrostatic interaction with negatively charged DNA *in vivo*. Whether this interaction with DNA results in peptide-induced inhibition of DNA replication or transcription *in vivo* remains to be determined. Although MtDef5_V1 retains its ability to bind to DNA at 6 and 12 μM, its lack of antibacterial activity is most likely due to its inability to enter into bacterial cells.

## Discussion

Plant defensins containing four disulfide bonds are a diverse group of AMPs with real potential for deployment as peptide antibiotics in agriculture. A phylogenetic analysis of 139 plant defensins has led to the classification of these defensins into eighteen distinct groups (van der Weerden and Anderson, 2013). A vast majority of these defensins fall into groups that exhibit antifungal activity against various fungal pathogens. To date, only one defensin in Group 1, one in Group 12, three in Group 13 and one in Group 15 have been shown to exhibit antibacterial activity. The bi-domain MtDef5 is a novel member of the defensin family present in the model legume *M. truncatula*. It was first identified as a highly potent antifungal defensin which exhibits antifungal activity at submicromolar concentrations against a broad range of fungi (Islam et al., 2017).

In this study, we have tested the antibacterial activity of MtDef5 and its two domains MtDef5A & MtDef5B against a Gram-negative bacterial pathogen *X. campestris* and a Gram-positive bacterial pathogen *C. michiganensis*. We have determined that MtDef5, MtDef5A, and MtDef5B inhibit the growth of *X. campestris* at micromolar concentrations. Surprisingly, MtDef5B was more potent than its parental bi-domain defensin MtDef5 as well as MtDef5A. MtDef5B, but not MtDef5A, showed greater antibacterial activity, with an MIC of 6 μM and an MBC of 12 μM compared to MIC value of 12 μM and MBC value of greater than 12 μM for MtDef5 and MtDef5A. This observation was quite surprising since MtDef5 exhibits greater antifungal activity than either MtDefA or MtDef5B (Islam et al., 2017). Amino acid sequences of MtDef5A and MtDef5B differ from each other in eight amino acids. Comparative sequence analysis shows that MtDef5A carries a net charge of +7, whereas MtDef5B carries a net charge of +8. Higher cationicity of MtDef5B may be a contributing factor for its more potent antibacterial activity than the antibacterial activity of MtDef5A. Hydrophobicity representing percentage of hydrophobic residues in a peptide is also an important parameter for antibacterial activity of AMPs including defensins. MtDef5A and MtDef5B carry 40% hydrophobic residues and thus hydrophobicity is unlikely to cause a significant increase in the antibacterial activity of MtDef5B. However, it is not clear as to why MtDef5B is more potent than its parent MtDef5. MtDef5 is more than twice the size of MtDef5B and its entry into *X. campestris* cells might be slower, thus delaying its binding to the intracellular targets and reducing its bactericidal activity. A more detailed study is needed to assess any potential difference in the kinetics of the uptake of MtDef5 and MtDef5B into bacterial cells.

MtDef5 and its two domains failed to inhibit growth of the Gram-positive bacterial pathogen *C. michiganensis* even at a concentration greater than 12 μM. *In vitro*, antibacterial activity of defensins might depend upon the structure of the bacterial cell envelope and surrounding polysaccharides. In Gram-positive bacteria, such as *C. michiganensis*, the cell wall includes the membrane and peptidoglycan layer. However, the cell wall of Gram-negative bacteria, such as *X. campestris*, contains an additional outer membrane. Thus, the lack of antibacterial activity against *C. michiganensis* may be due to the possibility that these peptides are unable to bind to the thick outer layer of peptidoglycan present in the cell wall of the Gram-positive *C. michiganensis* cells. On the other hand, these peptides must be able to bind to the outer layer of lipopolysaccharide and/or proteins present in the cell wall of the Gram-negative *X. campestris* cells. *Zea mays* defensin *Zm*ESR-6 showed stronger *in vitro* antibacterial activity against *C. michiganensis* than *X. campestris*; albeit with unknown MOA (Balandin et al., 2005). Thus, it will be informative to compare the amino acid sequence of this defensin with that of MtDef5B and identify putative sequence motifs that may be important for inhibitory activity of *Zm*ESR-6 against *C. michiganensis*. Antibacterial activity of MtDef5 and its two domains needs to be tested against other Gram-positive bacterial pathogens to determine if they lack antibacterial activity against all Gram-positive bacteria.

The γ-core motif (GXCX_3-9_C, where X is any amino acid), situated in the loop 5 flexible region between β strands 2 and 3, is conserved in all plant defensins (Kaur et al., 2011; Sagaram et al., 2012; De Coninck et al., 2013). This motif contains amino acids essential for the antifungal activity of a plant defensin. We have previously characterized the antifungal activity of the γ-core motif variant MtDef5_V1 containing mutations of the cationic amino acid residues (H36 and R37 in domain A and H93 and R94 in domain B) to alanine and determined that these residues of its two γ-core motifs were critical for the potent antifungal activity of MtDef5 (Islam et al., 2017). In this study, we found the MtDef5_V1 variant to have lost its antibacterial activity against *X. campestris* as well. Thus, the cationic residues present in the γ-core motifs of MtDef5A and MtDef5B are essential for the antibacterial activity of their parent MtDef5. We propose that the cationic residues are important for binding to the negatively charged site(s) of the lipopolysaccharide layer in the outer membrane and, in addition, to the negatively charged intracellular targets of *X. campestris*. It is also worth noting here that the nearly identical γ-core motifs of MtDef5A and MtDef5B serve as essential determinants of bacterial and fungal killing by their parent MtDef5.

In the present study, using the SG uptake assay, we showed that MtDef5 and MtDef5B permeabilize the plasma membrane of *X. campestris* cells suggesting membrane permeabilization is one of the contributing factors for the bacterial cell killing by these peptides. In addition to membrane disruption, we also observed distinct morphological changes induced by MtDef5, MtDef5_V1 variant, MtDef5A and MtDef5B in *X. campestris* cells. After 2 h of treatment with MtDef5, bacterial cells became spherical. In contrast, bacterial cells treated with MtDef5_V1 variant formed chain-like structures. As observed here for MtDef5-treated *X. campestris* cells, Gram-negative *Escherichia coli* cells treated with an antibacterial polymer also went from being rod-shaped to spherical and Gram-negative *Pseudomonas aeruginosa* cells treated with β-lactam antibiotics also switch from being rod shaped to spherical cells. However, the spherical cells remained viable (Young, 2008; Mukherjee et al., 2017). In contrast to MtDef5 treated *X. campestris* cells, MtDef5A treated cells showed shrinkage and some reduction in size, whereas MtDef5B caused significant disruption of cell integrity, leakage of cell contents and clumping. It should be noted that the morphological changes induced by these peptides were not observed in *X. campestris* cells challenged with isopropanol. It is noteworthy that the bi-domain defensin and its single domain counterparts are capable of inducing such strikingly different morphological changes in the bacterial cells suggesting perhaps that these sequence related defensin peptides likely operate via overlapping but not identical MOA. To our knowledge, this is the first example of a plant defensin that causes distinct morphological changes in *X. campestris*. Small nodule-specific cysteine-rich (NCR) peptides from *M. truncatula* have also been demonstrated to induce morphological changes in Gram-negative and Gram-positive bacterial pathogens (Tiricz et al., 2013).

Antibacterial peptides kill bacterial cells using multiple MOA. These include cell membrane permeabilization and translocation into the cell interior and interaction with intracellular targets (Shi et al., 2016). Confocal microscopy of *X. campestris* cells treated with fluorescently labeled MtDef5 and MtDef5B revealed that both peptides translocate across the cell wall, periplasmic space and plasma membrane and become dispersed in the cytosol of bacterial cells. These peptides likely have multiple targets in the bacterial cells. We first used membrane impermeant dye PI to assess the viability of *X. campestris* cells upon challenge with defensins. The uptake of PI into bacterial cells was visible within 3 h in *X. campestris* cells treated with MtDef5, MtDef5A, and MtDef5B, providing further evidence that these defensins disrupt the integrity of the cell membrane and affect cell viability. As expected, no uptake of PI was observed when *X. campestris* cells were challenged with the MtDef5 γ-core motif variant, MtDef5_V1, and these results confirm that MtDef5 and its two domains exert their antibacterial activity via membrane damage. The MOA of NCR247 and NCR335 defensin-like peptides against *Sinorhizobium meliloti* was similarly attributed to damage to the integrity of both the inner and outer bacterial membranes, leading to altered membrane potential and cell death (Tiricz et al., 2013).

In addition to membrane-permeabilizing/disrupting properties, AMPs translocate across the disrupted membrane and into the cytoplasm to interact with intracellular targets such as nucleic acids and protein synthesis complex, and thereby interfere with cell physiological processes. Confocal microscopy of *X. campestris* cells treated with fluorescently labeled MtDef5 and MtDef5B revealed that both peptides were internalized and localized throughout the cytosol of bacterial cells.

The co-localization of MtDef5 and MtDef5B with SG revealed that these peptides interact with nucleic acids. This hypothesis was further investigated using an electrophoretic mobility shift, or gel retardation, assay which revealed that MtDef5 more strongly bound to bacterial genomic DNA and plasmid DNA at all concentration*s* than MtDef5A and MtDef5B. Stronger binding of MtDef5 to DNA is expected since it carries a higher net positive charge than its either domain and can be electrostatically attracted by the negatively charged DNA even at lower concentrations of MtDef5. In contrast to MtDef5 which binds to DNA at a low concentration of 3 μM, its variant with the H36A, R37A, H93A, and R94A substitutions in its γ-core motifs exhibits markedly reduced binding at this concentration. This observation reveals the importance of the cationic residues present in the γ-core motif for interaction of MtDef5 with DNA. The AMP melittin has also been previously shown to translocate into the cytoplasm of *X. oryzae* and interact with DNA (Shi et al., 2016). It is plausible that the process of DNA replication, transcription or both could be affected by MtDef5 and its two domains. Further studies are needed to determine if these peptides inhibit transcription and/or translation *in vitro* or *in vivo.* In future studies, effects of MtDef5 and MtDef5B on *X. campestris* bacterial cells and their ability to cause disease will be examined *in planta*.

Based on the results in the present study, we propose a multi-step mechanism for the antibacterial action of MtDef5 and its two domains against *X. campestris* that involves: (1) an initial interaction with outer surface of bacteria; (2) permeabilization of bacterial membranes; (3) internalization into the cytoplasm; (4) interaction with bacterial DNA and possible inhibition of DNA replication and/or transcription; (5) interaction with cytoplasmic targets.

*X. campestris* pv. *campestris* (Xcc), a Gram-negative hemibiotrophic pathogen, is the causal agent of Xanthomonas wilt disease which affects plants of the family Brassicaceae and is ranked among “Top 10” bacterial pathogens of economic and scientific importance (Mansfield et al., 2012). In the absence of host plant resistance to the Xanthomonas wilt disease caused by *X. campestris*, constitutive expression of either MtDef5 and/or MtDef5B in transgenic host plants could provide resistance to this pathogen. Genetic engineering of Xanthomonas wilt resistant crop varieties could complement conventional breeding by allowing the bottlenecks of breeding for developing resistant varieties to be overcome.

## Author Contributions

SV, KI, and DS designed the research, analyzed the data, and wrote the paper. SV, KI, and EH performed the research.

## Conflict of Interest Statement

The authors declare that the research was conducted in the absence of any commercial or financial relationships that could be construed as a potential conflict of interest.
