# Supplementary figures and images for: Modes of Action of a Bi-domain Plant Defensin MtDef5 Against a Bacterial Pathogen Xanthomonas campestris
Source: Front Microbiol. 2018 May 16;9:934. doi: 10.3389/fmicb.2018.00934 (PMC5964164; doi:10.3389/fmicb.2018.00934)

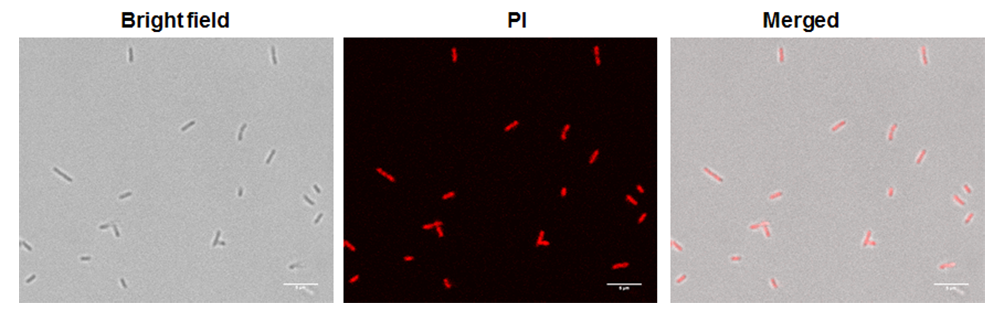

Supplement: Supplementary file 1 [file Image_1.TIF]
